# Supplementary material for: stuart: an R package for the curation of SNP genotypes from experimental crosses
Source: G3 (Bethesda). 2022 Aug 24;12(11):jkac219. doi: 10.1093/g3journal/jkac219 (PMC9635635; doi:10.1093/g3journal/jkac219)
Supplement: jkac219_Supplementary_Material_Legends [file jkac219_supplementary_material_legends.docx]

**Supplementary data**

**Supplementary Table 1**

Format of the phenotype data. First column: individual's number. Second column: individual's sex. Following columns: traits and covariates (individual's age, phenotype)

**Supplementary Figure 1**

Distribution of the quantitative phenotypes analyzed in the three datasets.

**Supplementary Figure 2**

Analysis of datasets 2 and 3 illustrating the expansion of the estimated genetic maps. Non-polymorphic markers and markers with more than 50% missing genotypes were excluded to avoid excessive calculation time. A, B: comparison of the known marker map (left) and the genetic map estimated from observed RF (right), as calculated by est.map() and represented by plotMap() functions of R/qtl in dataset 2 (A) and dataset 3 (B). Lines connect the positions of each marker in the two maps. The estimated map is considerably expanded because of multiple genotype inconsistencies. C, D: distribution of the ratio between estimated and known distance between adjacent markers. Markers with known and calculated distances below 1cM were removed as they may lead to extremely small or large ratios. The expansion of the estimated map leads to a distribution tail of high ratios. The y-axis is in logarithmic scale. 51% of the markers in the dataset 2 (C) and 16% of the markers in the dataset 3 (D) have a ratio above 5 (dashed line).

**Supplementary Figure 3**

Analysis of datasets 2 and 3 illustrating the identification of narrow LOD-score peaks. A, B: output of the scanone() function of R/qtl in the datasets 2 (A) and 3 (B) showing the identification of two narrow suggestive peaks. C: peak p1 from dataset 1 (see Figure 1C) is located on a single marker (mUNC050096588, red tick) with non-Mendelian proportions. Peak p2 from dataset 1 shows the same pattern. D: genotypes at mUNC050096588. HM1 and HM2: homozygotes; HT: heterozygotes; NA: missing genotypes. E: peak p3 from dataset 2 is located on a pseudomarker adjacent to a marker with non-Mendelian proportions (SNT111392585, red tick). Peak p4 from dataset 3 shows the same pattern. F: genotypes at SNT111392585.

**Supplementary Figure 4**

Analysis of the estimated genetic map in datasets 2 (A) and 3 (B) after curation of genotyping data by stuart. Refer to Supplementary Figure 2 for comparison with original data. The estimated marker maps are now consistent with the known marker maps with similar genome length despite local contractions and expansions (the ratio between the calculated and the known length of the genome is 1.00 for dataset 2 and 0.96 for dataset 3). The ratios between estimated and known distance between adjacent markers in dataset 2 (C) and dataset 3 (D) are now normally distributed with a mean=1.27 and a sd=0.77 for dataset 2 and a mean=1.24 and a sd=0.61 for dataset 3. The x-axis is in logarithmic scale.

**Supplementary Figure 5**

Analysis of the LOD score curve after curation of genotyping data by stuart in datasets 2 (A) and 3 (B). Refer to Supplementary Figure 3 for comparison with original data. Significance thresholds are much lower than before curation. One peak in dataset 2 is significant at P<0.05 (plain line) and none of the peaks observed before data curation (Supplementary Figure 3) were confirmed after curation with stuart. Dotted line: P=0.1. Dashed line: P=0.63 (genome-wide significance computed by data permutation).
