# Supplementary figures and images for: stuart: an R package for the curation of SNP genotypes from experimental crosses
Source: G3 (Bethesda). 2022 Aug 24;12(11):jkac219. doi: 10.1093/g3journal/jkac219 (PMC9635635; doi:10.1093/g3journal/jkac219)

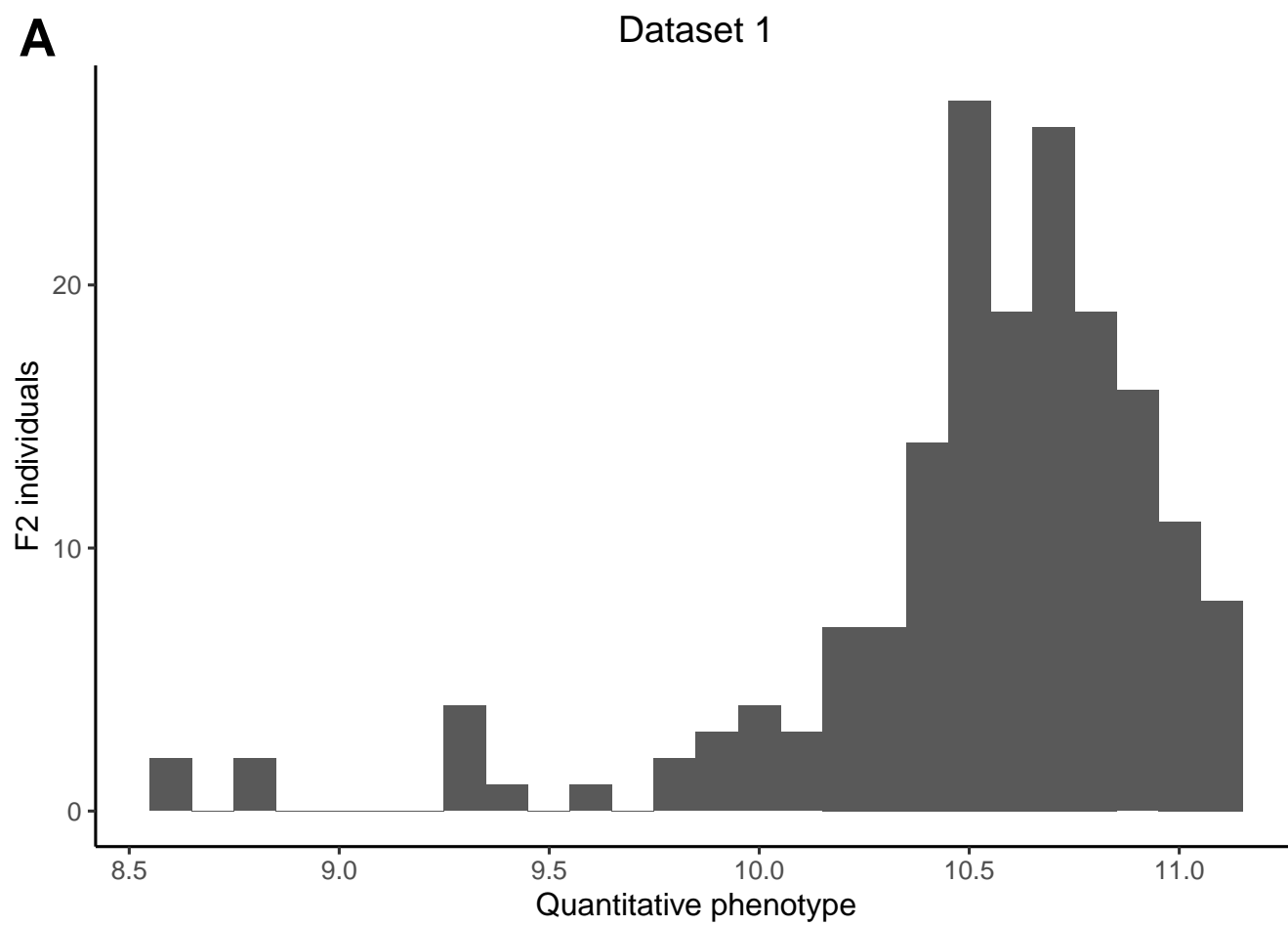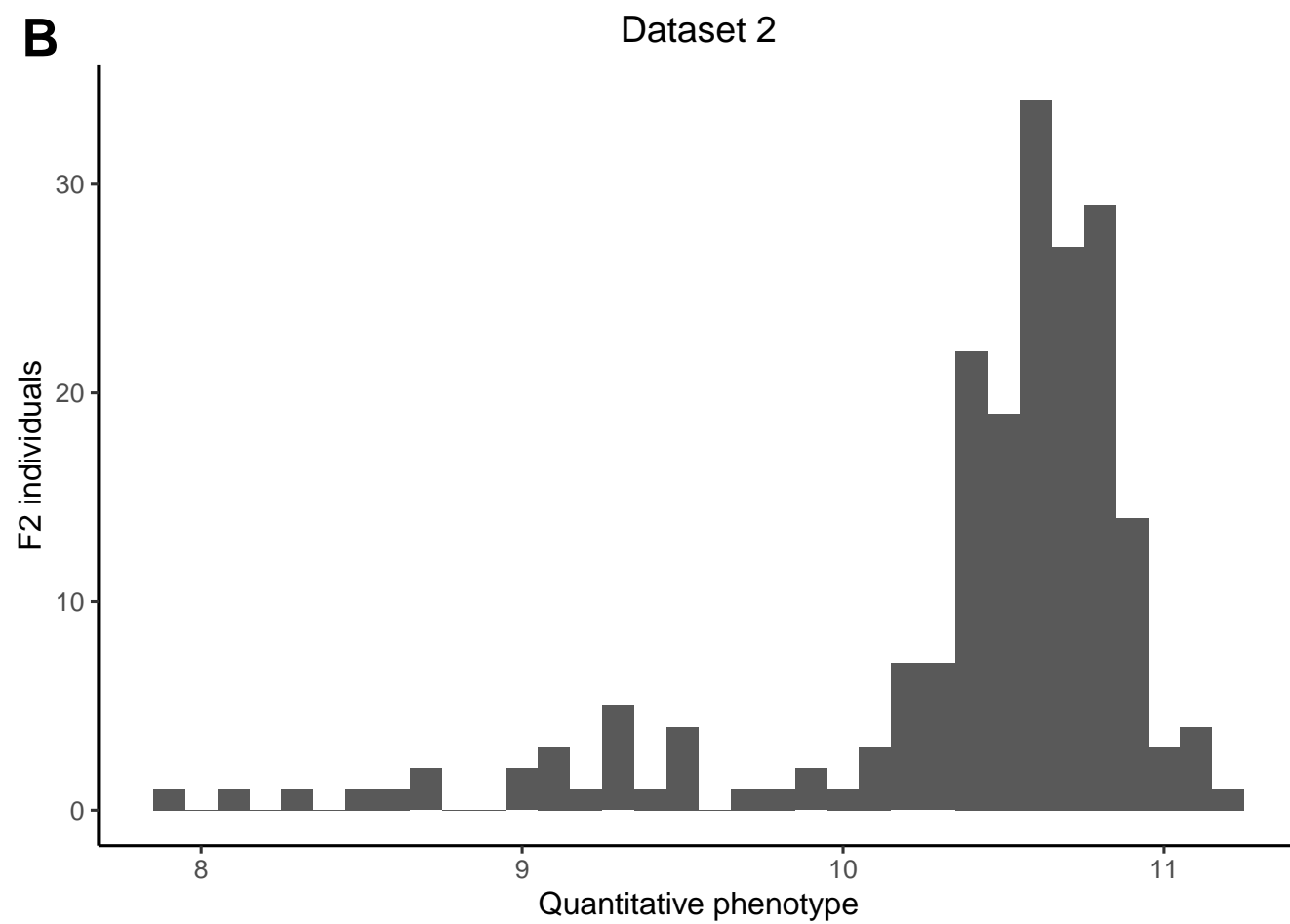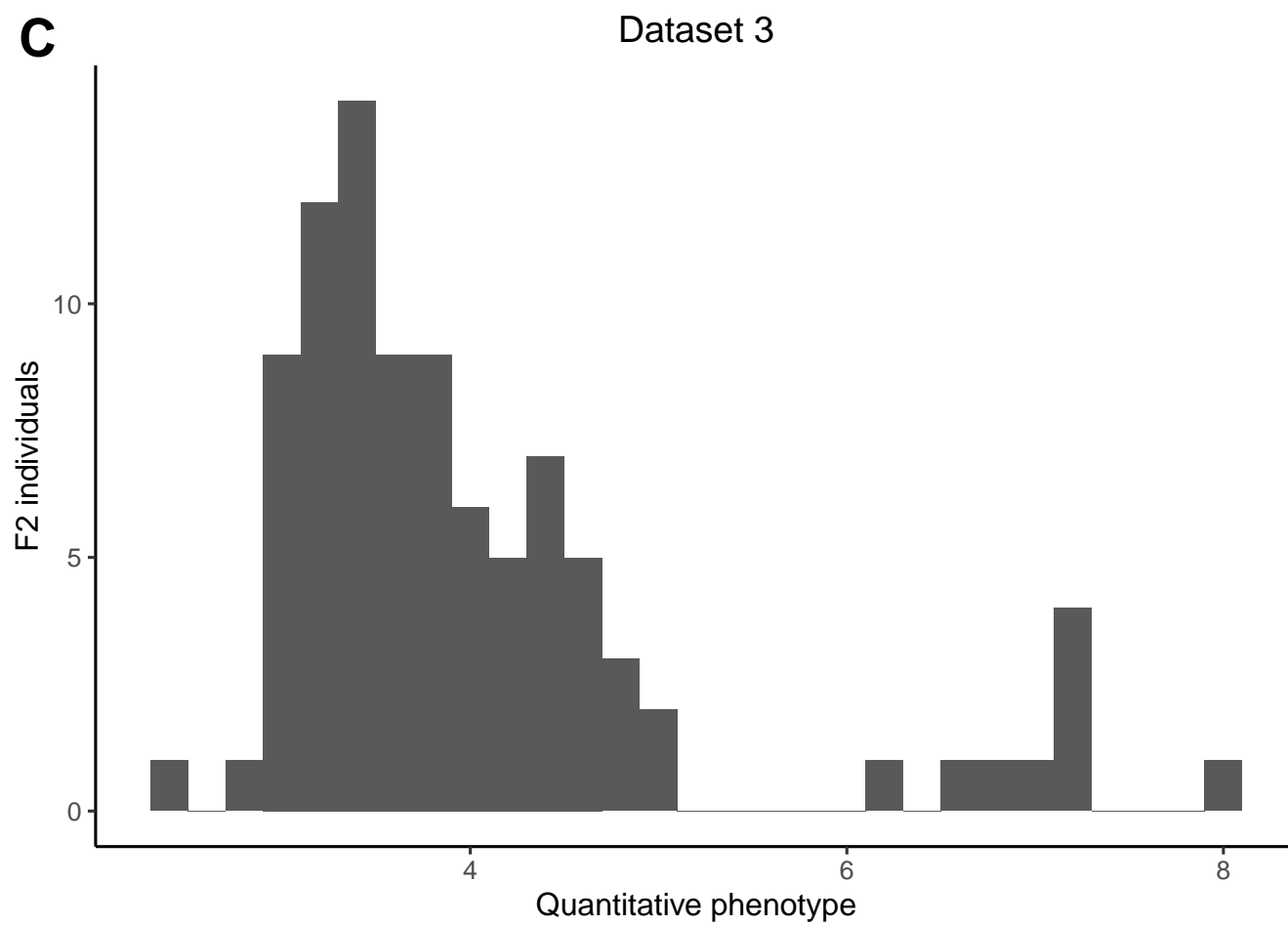

Supplement: jkac219_Supplementary_Figure_1 [file jkac219_supplementary_figure_1.pdf]

**A**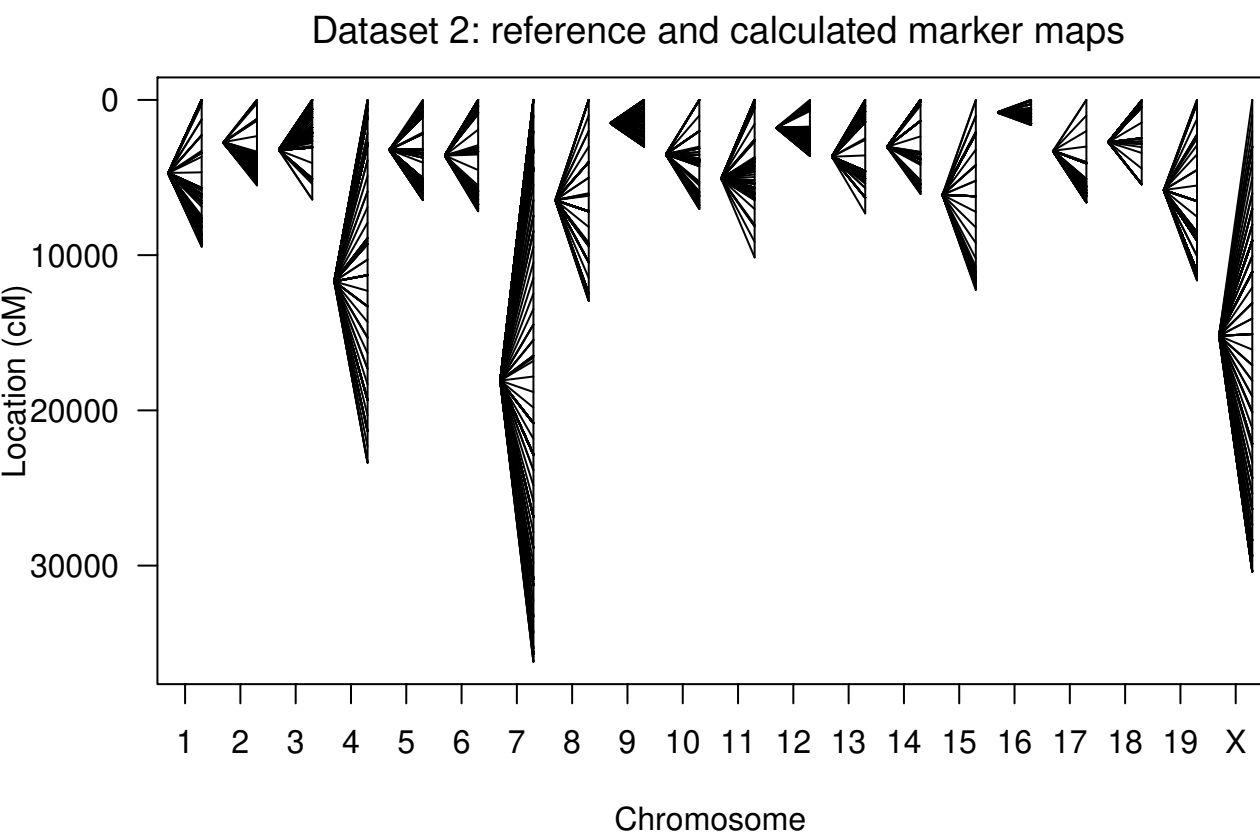**B**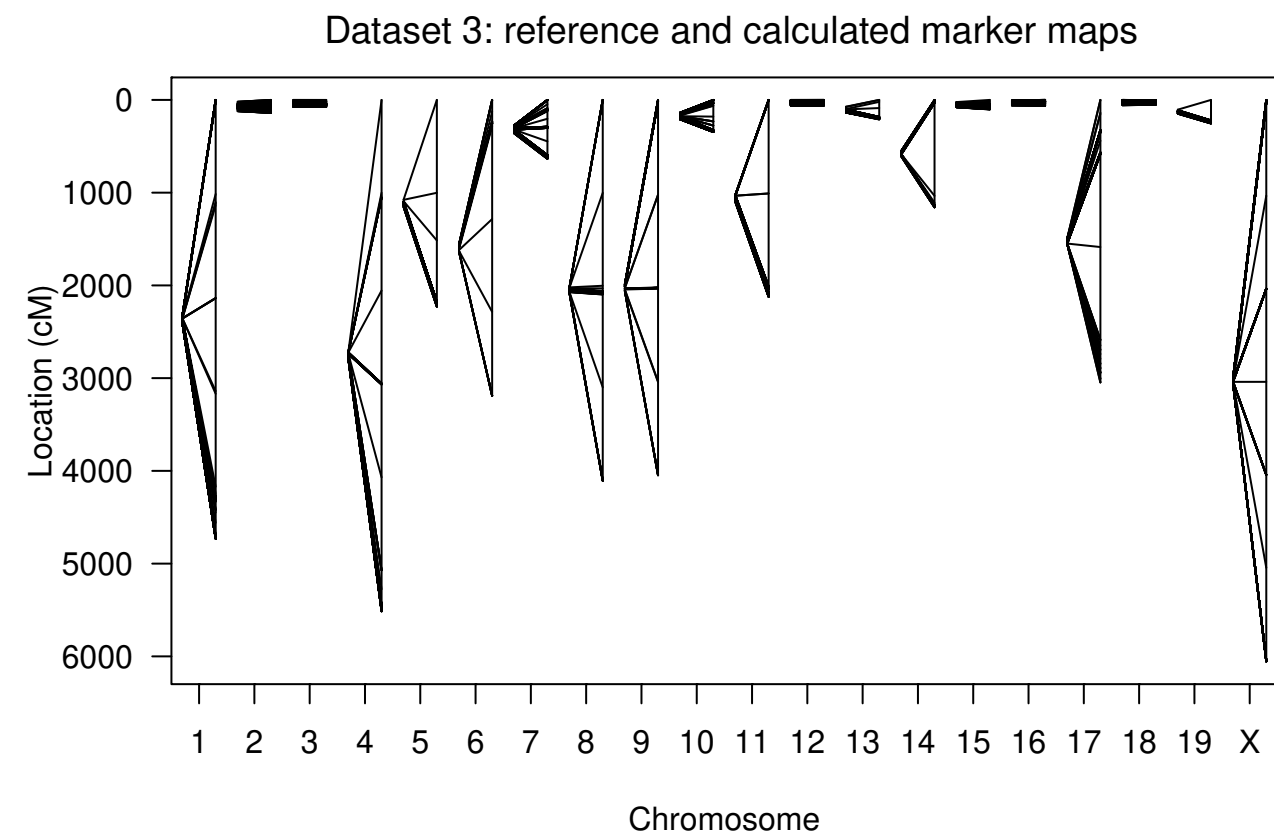**C**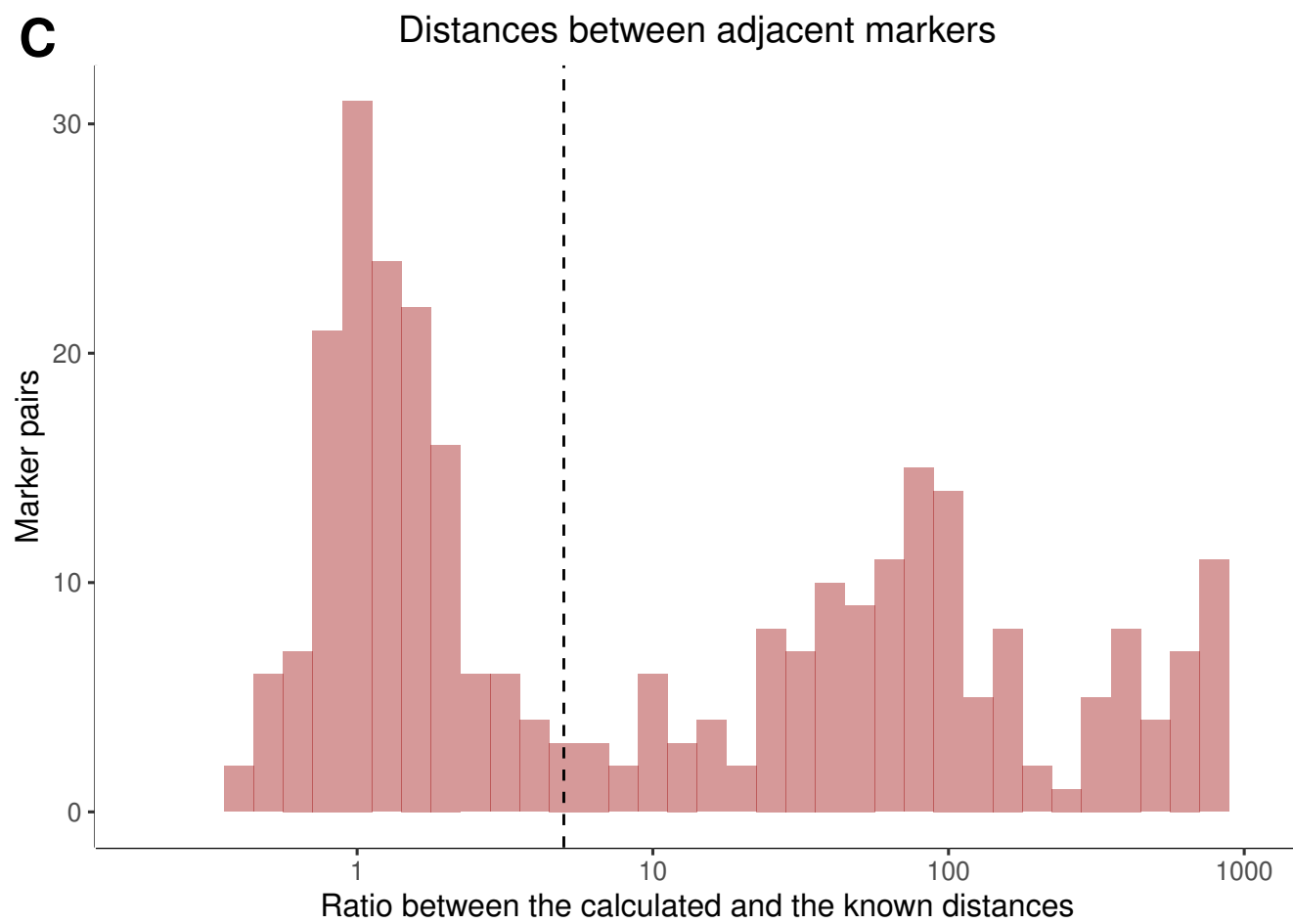**D**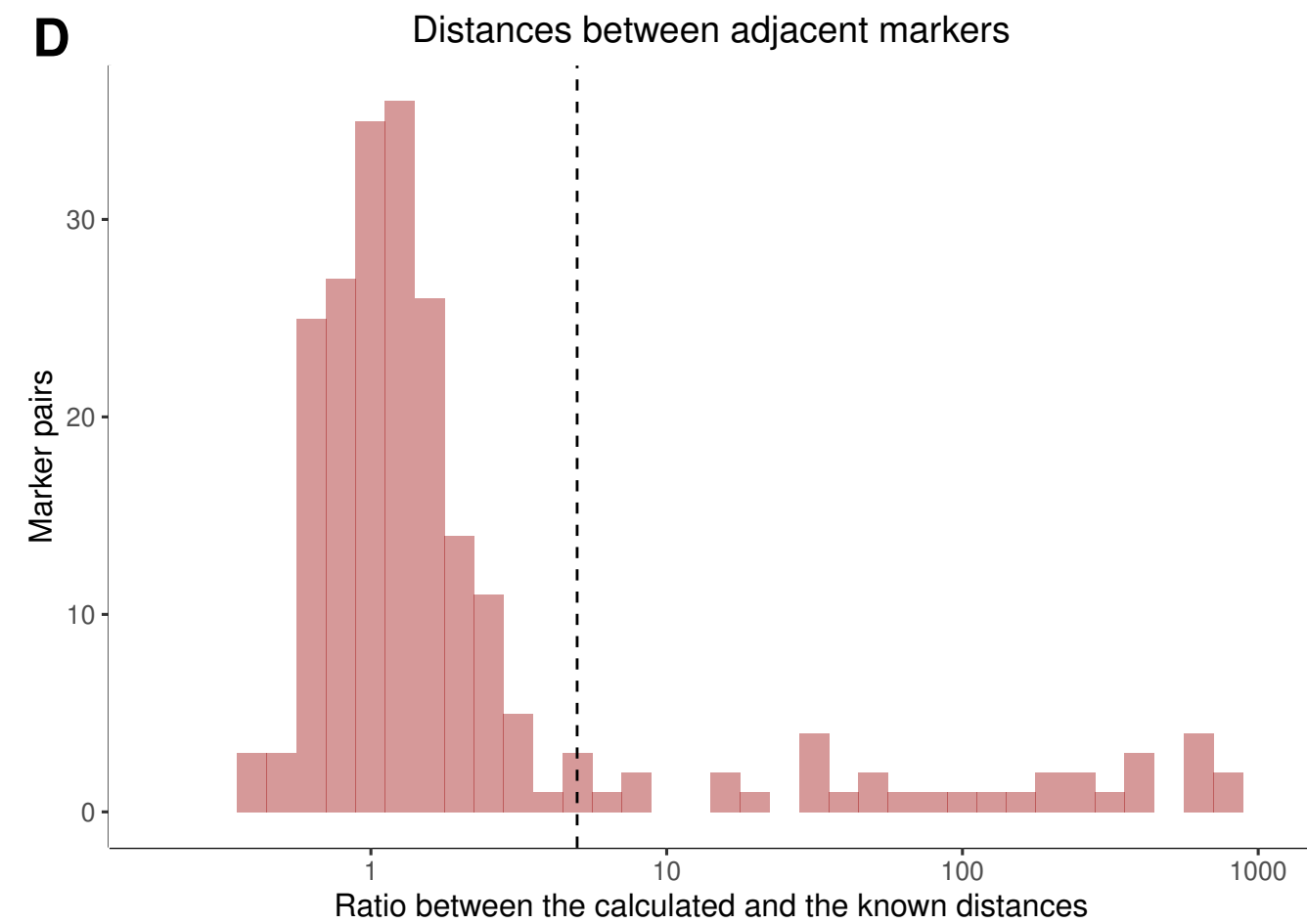

Supplement: jkac219_Supplementary_Figure_2 [file jkac219_supplementary_figure_2.pdf]

**A**

Dataset 2: genome scan

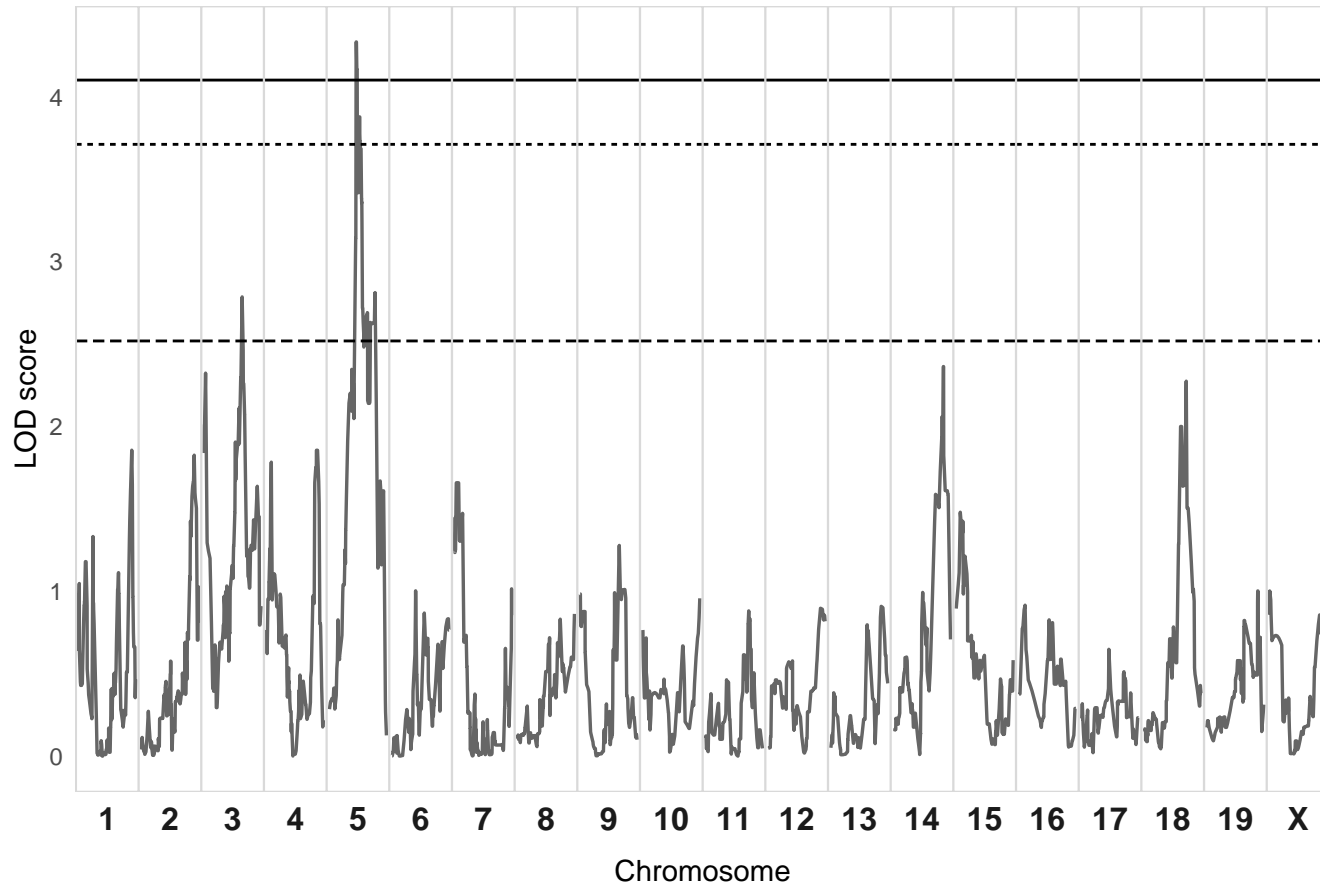**B**

Dataset 3: genome scan

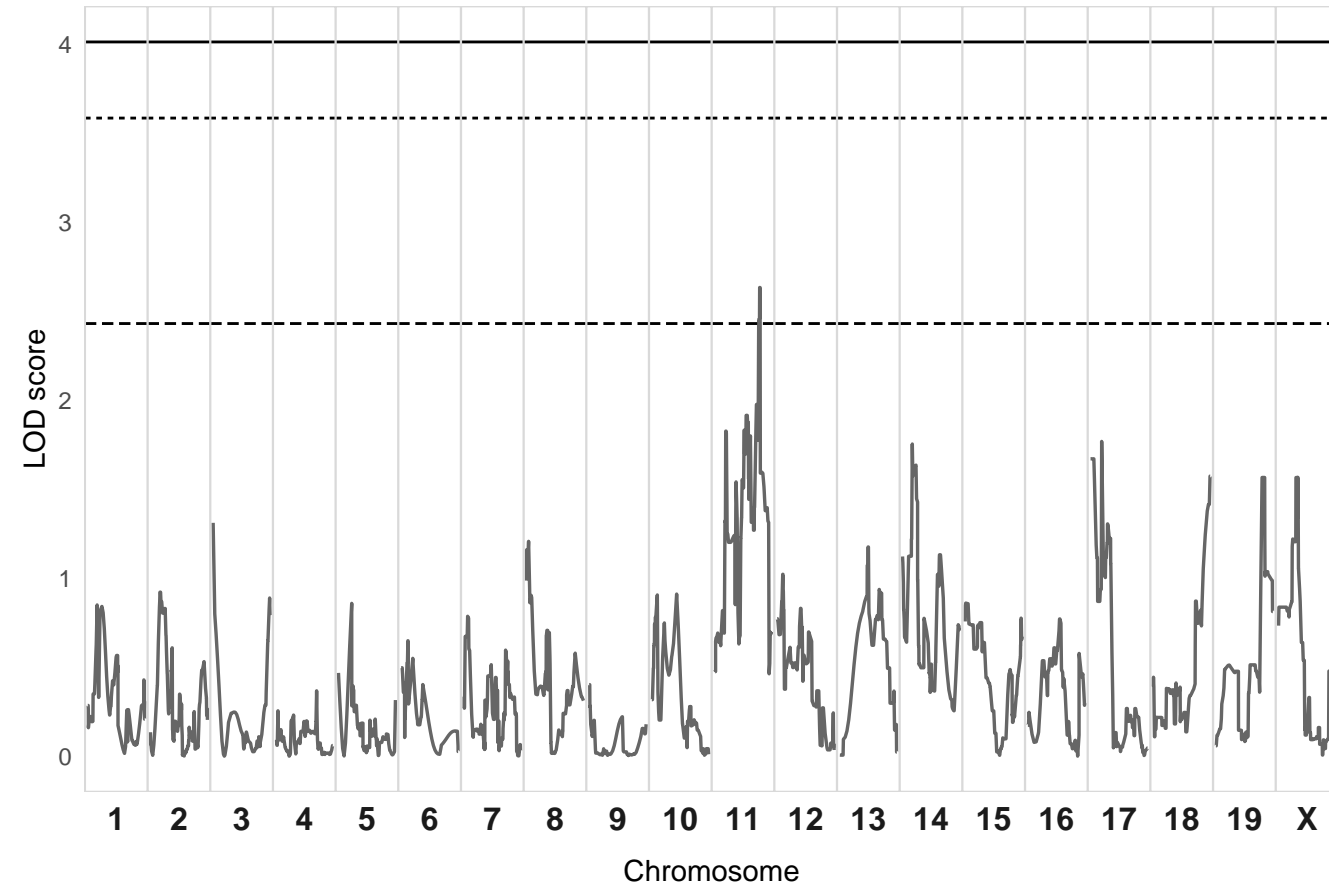

Supplement: jkac219_Supplementary_Figure_5 [file jkac219_supplementary_figure_5.pdf]
