# Supplementary material for: stuart: an R package for the curation of SNP genotypes from experimental crosses
Source: G3 (Bethesda). 2022 Aug 24;12(11):jkac219. doi: 10.1093/g3journal/jkac219 (PMC9635635; doi:10.1093/g3journal/jkac219)
Supplement: jkac219_Supplementary_Figure_3 [file jkac219_supplementary_figure_3.pdf]

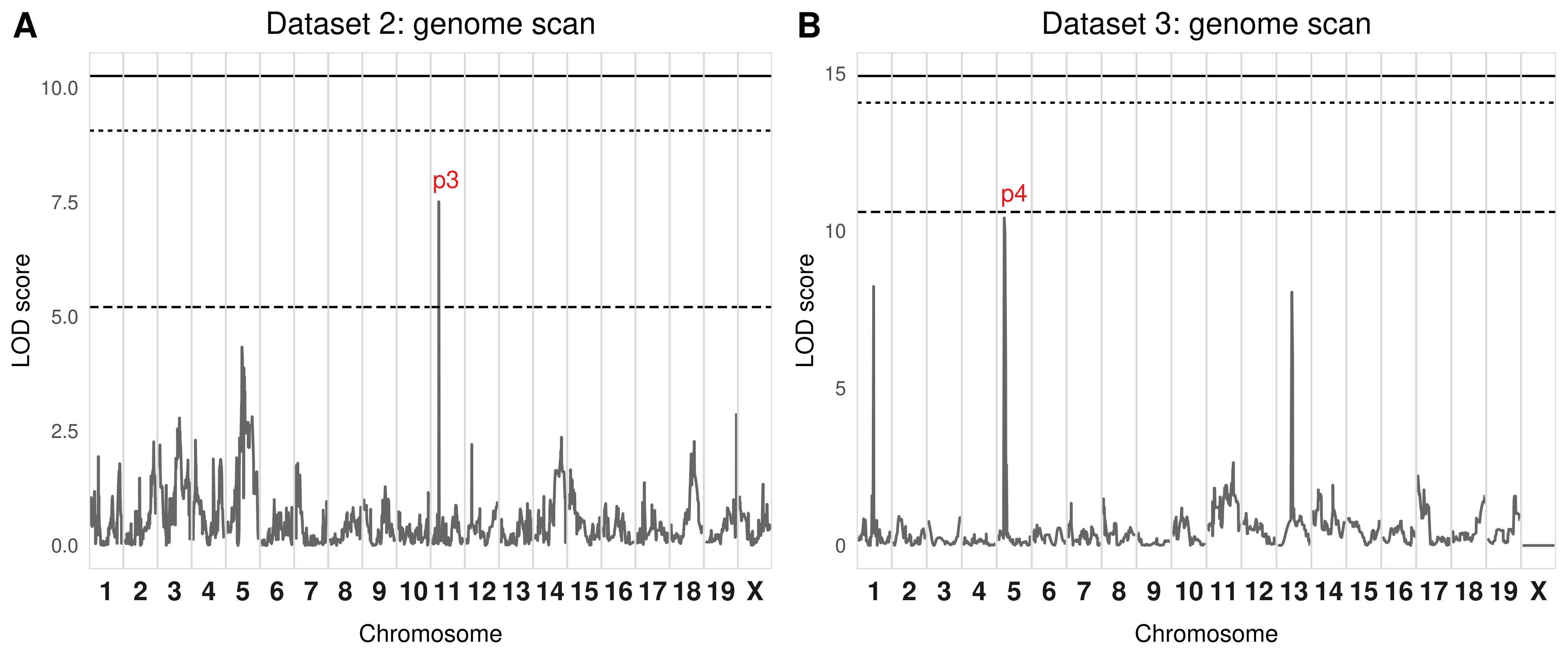

Peak on a single marker (red) with non-Mendelian proportions (peaks 1 and 2)

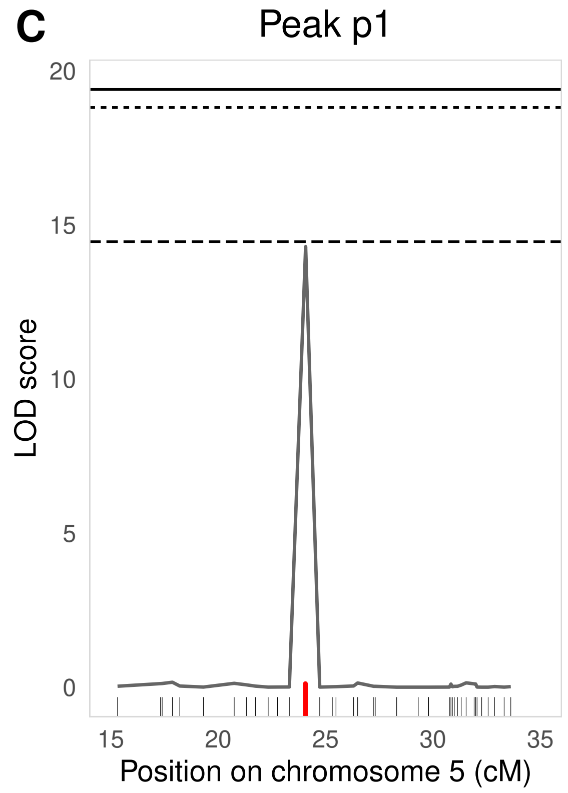

D Genotype at mUNC050096588 (red tick)

marker	chr	pos	allele 1	allele 2	n HM1	n HM2	n HT	n NA
mUNC050096588	5	24.069	A	G	0	0	176	0

Peak on a peudomarker adjacent to a marker (red) with non-Mendelian proportions (peaks 3 and 4)

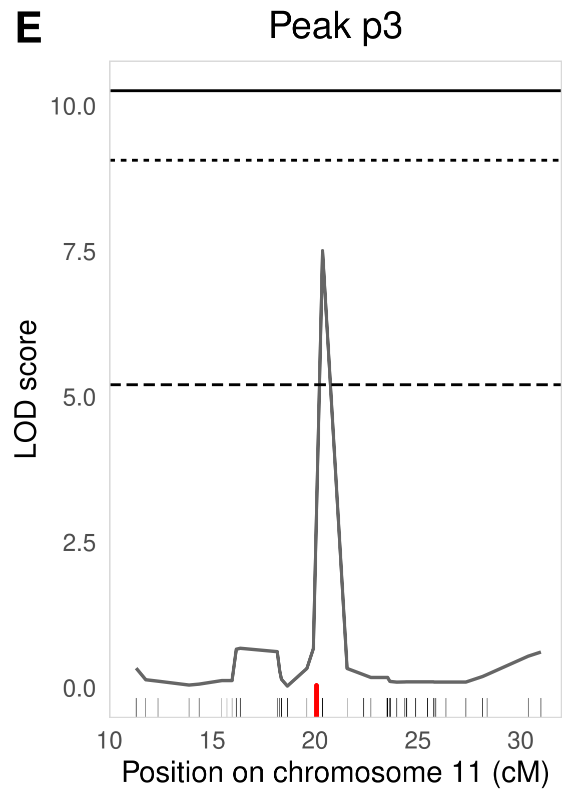

F Genotype at SNT111392585 (red tick)

marker	chr	pos	allele 1	allele 2	n HM1	n HM2	n HT	n NA
SNT111392585	11	19.917	T	C	0	90	1	3
