# Supplementary material for: stuart: an R package for the curation of SNP genotypes from experimental crosses
Source: G3 (Bethesda). 2022 Aug 24;12(11):jkac219. doi: 10.1093/g3journal/jkac219 (PMC9635635; doi:10.1093/g3journal/jkac219)
Supplement: jkac219_Supplementary_Figure_4 [file jkac219_supplementary_figure_4.pdf]

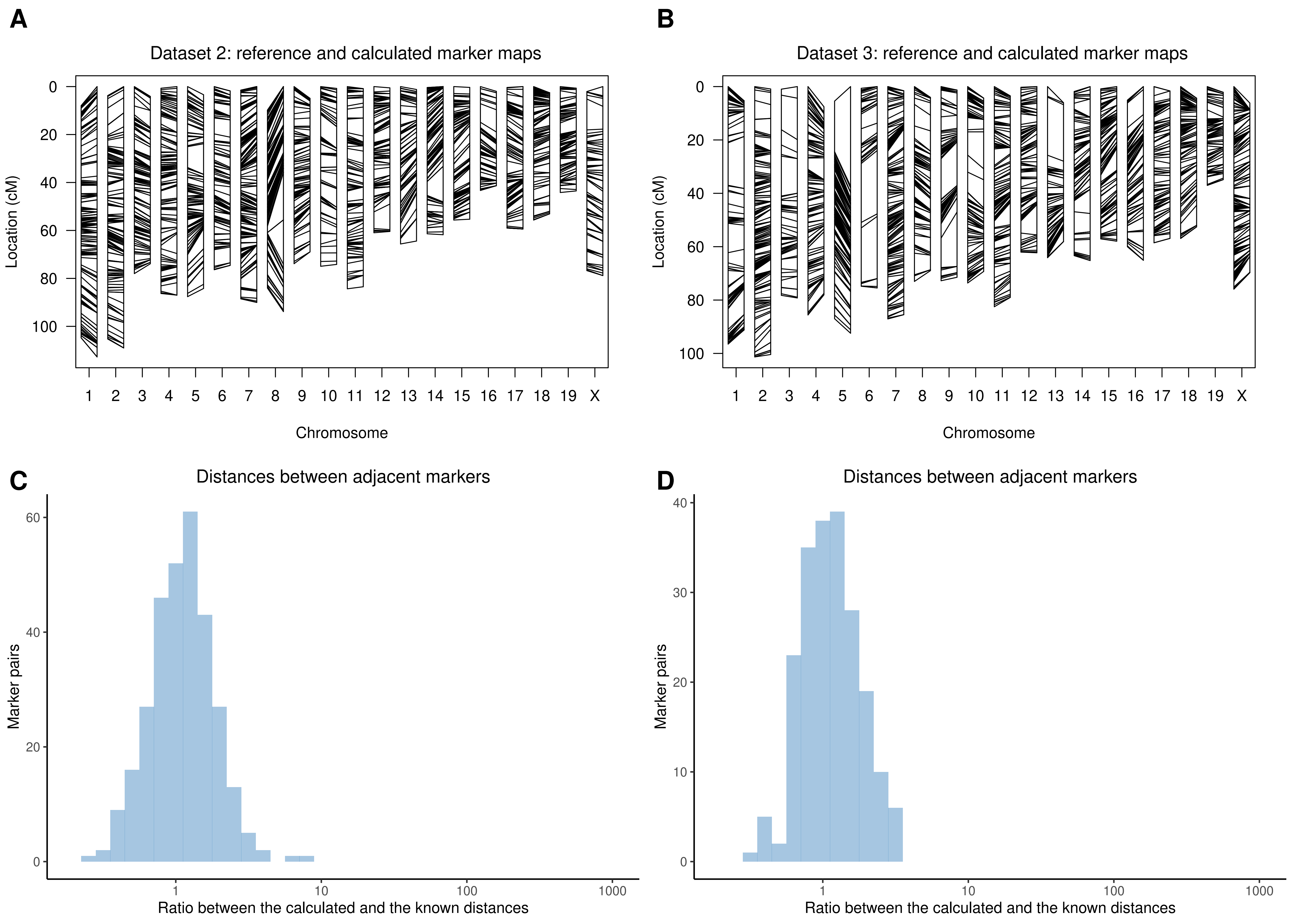
A
Dataset 2: reference and calculated marker maps
Location (cM)
Chromosome
B
Dataset 3: reference and calculated marker maps
Location (cM)
Chromosome
C
Distances between adjacent markers
Marker pairs
Ratio between the calculated and the known distances
D
Distances between adjacent markers
Marker pairs
Ratio between the calculated and the known distances
